# Head‐to‐head prospective comparison of diagnostic accuracy of preoperative ultrasonography and MRI in patients undergoing surgery for deep endometriosis using #Enzian classification

**DOI:** 10.1002/uog.70298

**Published:** 2026-08-06

**Authors:** B. Pete, K. Turtóczki, A. Bokor, I. Kalina, I. Madár, L. Lipták, J. Rigó, N. Ács, G. Szabó

**Affiliations:** ^1^ Department of Obstetrics and Gynecology, Faculty of Medicine Semmelweis University Budapest Hungary; ^2^ Department of Radiology, Medical Imaging Centre Semmelweis University, Faculty of Medicine Budapest Hungary; ^3^ Department of Clinical Studies in Obstetrics and Gynecology, Faculty of Health Sciences Semmelweis University Budapest Hungary

**Keywords:** diagnostic accuracy, endometriosis, head‐to‐head comparison, magnetic resonance imaging, transvaginal sonography, ultrasound

## Abstract

**Objective:**

To perform a head‐to‐head comparison of the diagnostic performance of ultrasonography (US) *vs* magnetic resonance imaging (MRI) for the preoperative detection of endometriosis using the #Enzian classification system.

**Methods:**

This was a prospective observational study of postmenarchal premenopausal women who underwent laparoscopy between 1 April 2021 and 31 May 2024 in a tertiary referral center for clinical suspicion of endometriosis. All women underwent both transvaginal and transabdominal US imaging, according to the International Deep Endometriosis Analysis (IDEA) protocol, as well as MRI evaluation. The sonologist and the radiologist were blinded to each other's results. The diagnostic performance of US and MRI were determined for the following #Enzian compartments: O (ovarian endometriosis), T (adhesions at the level of the tubo‐ovarian unit), A (deep endometriosis (DE) in the vagina, rectovaginal septum and torus uterinus), B (DE in the uterosacral ligament, cardinal ligament, parametrium and pelvic sidewall), C (DE in the rectosigmoid bowel), FI (lesions in other intestinal locations), FB (urinary bladder involvement), FU (ureteric involvement) and FO (lesions in other extragenital locations). The sensitivity and specificity were compared between imaging modalities using McNemar's test. Concordance between preoperative imaging and surgical assessment of #Enzian severity grade for endometriotic lesions/adhesions in #Enzian compartments O, T, A, B and C was assessed.

**Results:**

In total, 463 women who underwent laposcopy for diagnosis and excisional treatment of endometriosis were enrolled in the study. For the detection of endometriotic lesions/adhesions in #Enzian compartments O_left_, O_right_, T_left_, T_right_, A, B_left_, B_right_, C, FI, FU, FB and FO, US had a sensitivity of 97.52%, 97.22%, 85.15%, 85.26%, 92.66%, 88.81%, 86.79%, 95.79%, 75.00%, 76.19%, 83.72% and 60.00%, respectively, and a specificity of 96.77%, 97.73%, 96.63%, 94.79%, 91.02%, 96.66%, 96.92%, 95.60%, 98.77%, 97.96%, 99.05% and 99.56%, respectively. For MRI, the corresponding values for sensitivity were 95.04%, 94.44%, 77.23%, 74.74%, 90.37%, 88.06%, 90.57%, 94.21%, 83.93%, 71.43%, 74.42% and 90.00%, and those for specificity were 95.89%, 95.75%, 92.70%, 89.86%, 93.47%, 96.05%, 94.96%, 94.14%, 97.05%, 99.10%, 98.57% and 98.90%. US had significantly higher specificity in #Enzian compartment T on both the left (*P* = 0.0336) and right (*P* = 0.0153) sides compared with MRI, but no other comparisons of sensitivity or specificity between US and MRI showed statistically significant differences. US showed higher exact concordance with surgical assessment in severity grading for lesions/adhesions in the adnexa than did MRI (O_left_, 96.3% *vs* 94.8%; O_right_, 97.2% *vs* 94.6%; T_left_, 93.9% *vs* 89.0%; T_right_, 92.6% *vs* 86.7%). For DE nodules, US showed a 3.1 percentage point higher exact concordance with laparoscopy compared with MRI in #Enzian compartment C (94.0% *vs* 90.9%).

**Conclusions:**

US evaluation following the IDEA protocol has significantly higher specificity compared with that of MRI for pelvic adhesions in #Enzian compartment T. However, US and MRI have similarly high sensitivity and specificity for diagnosing DE in all relevant #Enzian compartments. © 2026 The Author(s). *Ultrasound in Obstetrics & Gynecology* published by John Wiley & Sons Ltd on behalf of International Society of Ultrasound in Obstetrics and Gynecology.

## INTRODUCTION

Endometriosis is a chronic disease affecting 10–12% of women of reproductive age worldwide. It has a significant impact on patients' quality of life and is a leading cause of infertility. Although endometriosis is characterized by painful symptoms, such as dysmenorrhea, dyschezia, dysuria and dyspareunia, the lack of adequate diagnostic methods meant that, until recently, the presence and extent of disease were detected only during surgery in most cases. Until recently, diagnostic laparoscopy was considered as the gold standard for detection. The time from first symptom to diagnosis may be as long as 7–10 years^1^.

In order to reduce this diagnostic delay, many efforts have been made over the past two decades to develop different diagnostic techniques for endometriosis. Two key imaging modalities have emerged in this pursuit: ultrasonography (US) and magnetic resonance imaging (MRI)^2^. The standardized approach for gynecological US examination is summarized in the International Deep Endometriosis Analysis (IDEA) group consensus opinion^3^. Guidelines for the MRI examination of pelvic endometriosis were established by the European Society of Urogenital Radiology (ESUR) and the Society of Abdominal Radiology^4^, ^5^. Expert sonologists/sonographers and radiologists can detect endometriosis with high accuracy using US and MRI, respectively. The updated European Society of Human Reproduction and Embryology guideline recommends using US or MRI in the diagnostic work‐up for endometriosis^6^.

However, objective comparison of the effectiveness of different diagnostic methods in various anatomical localizations is possible only if both the preoperative diagnosticians and surgeons use the same classification system. The updated #Enzian classification has been developed and validated for this purpose^7^, ^8^, ^9^. A recent international intersociety consensus statement found strong agreement that imaging using either transvaginal ultrasonography (TVS) or MRI, in combination with the #Enzian classification, can reliably describe pelvic deep endometriosis (DE), ovarian endometriosis and adhesions, but is less accurate in cases of parametrial involvement^10^.

To the best of our knowledge, comparative studies of the preoperative diagnostic efficacy of US and MRI using the #Enzian classification have not yet been performed. The aim of this study was to perform a head‐to‐head comparison of the diagnostic performance of US *vs* MRI for the preoperative detection of endometriosis using the #Enzian classification.

## METHODS

### Study setting

This prospective, single‐center, observational, paired, head‐to‐head comparative diagnostic accuracy study was conducted between 1 April 2021 and 31 May 2024 in a European tertiary referral center for endometriosis (Department of Obstetrics and Gynecology, Semmelweis University, Budapest, Hungary). Inclusion criteria were postmenarchal and premenopausal non‐pregnant women over 18 years of age who exhibited clinical signs and symptoms of endometriosis (severe dysmenorrhea, cyclic dyschezia, dysuria, deep dyspareunia and/or chronic pelvic pain) and were selected for surgery. Exclusion criteria were the presence of any malignant pelvic tumor, refusal to undergo surgery and patients who declined to give consent to participate in the study. During preoperative diagnostic work‐up, both US and MRI were performed on all consecutively included patients. The time interval between US and MRI was not longer than 30 days, and the interval between the first imaging examination and surgery was not longer than 60 days. The scheduling of imaging examinations depended on the availability of examinations and, in the case of US, on the timing of menstrual bleeding. We did not perform scheduled TVS during expected menstrual bleeding. The sonologist and the radiologist were blinded to each other's results. The surgeon was informed about both results at the same time, but the indication for surgery had already been determined prior to the imaging examinations. Histological examination of the specimens obtained by laparoscopy was the reference standard for the diagnosis of endometriosis.

Clinical and demographic characteristics of the participating women were recorded, including age, body mass index, gravidity, parity, preoperative symptoms (dysmenorrhea, dyschezia, dysuria, dyspareunia, chronic pelvic pain, infertility, constipation, diarrhea, rectal bleeding and/or hematuria) and history of pelvic surgery.

According to the study of Guerriero *et al*.^11^, to obtain a statistically significant difference in sensitivity and specificity between 80% and 95%, considering a Type‐I error rate of 0.05 and a Type‐II error rate of 0.05, at least 150 patients should be recruited. The last meta‐analysis comparing the diagnostic accuracy of TVS *vs* MRI for DE in all locations, which included studies conducted prior to the publication of the IDEA approach and the ESUR guideline, included the data of 424 patients^12^. Therefore, we decided to set the sample size for our study at a minimum of 450 patients.

This observational study was approved by the national Human Research Ethics Committee (IV/9974‐12021/EKU). Only participants who provided written informed consent for participation in the study were included.

### Ultrasonography

All pelvic US examinations were performed by one of two gynecologists (G.S., I.M.) experienced in the US diagnosis of endometriosis (each had performed over 5000 TVS scans), using either a HERA W9 (Samsung, Seoul, Republic of Korea) ultrasound system equipped with a 3–12‐MHz transvaginal probe or a HERA W10 (Samsung) ultrasound system equipped with a 2–10‐MHz transvaginal probe with three‐dimensional functionality. A transabdominal probe (1–8‐MHz volume transducer on both machines) was used for evaluation of the abdominal wall, diaphragm and bowel cranial to the sigmoid colon and for assessment of the kidneys. The sonologists were blinded to the preoperative clinical findings.

Patients presenting with possible endometriosis were examined using TVS and transabdominal ultrasonography, according to the recommendations of the IDEA group^3^. To analyze the greatest diameter of the suspected endometriotic lesions, the dimensions of the lesions were recorded in three orthogonal planes using B mode. Additionally, the location, size and extent of all lesions were described using the #Enzian classification system.

#Enzian compartment P (peritoneum) was omitted because, usually, it can be assessed only during surgery^7^. Moreover, #Enzian compartment FA (adenomyosis) was also not evaluated in this study because hysterectomy was performed in a negligible proportion of cases, meaning that histological confirmation of adenomyosis was not always available.

Ovarian endometriosis (#Enzian compartment O) was defined based on the US characteristics of endometrioma in premenopausal women, such as ground‐glass echogenicity or hypoechogenic content of the cyst, presence of one to four locules and no solid parts^13^. Severity was graded according to the sum of the maximum diameters of all ovarian endometriotic lesions as follows: Grade 0, no lesion; Grade 1, < 3 cm; Grade 2, 3–7 cm; and Grade 3, > 7 cm.

**Figure 1:**
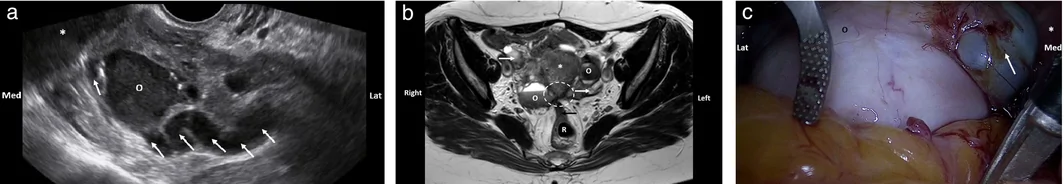
Endometriosis in #Enzian compartment T (tubo‐ovarian unit) in patient undergoing surgery. (a) Transvaginal ultrasound image in oblique view for the adnexa, with transducer in left lateral fornix. Typical endometrioma (unilocular cyst with ground‐glass echogenicity) is visible in left ovary (O) and hydrosalpinx (serpentiform unilocular hypoechogenic structure) (white arrows) is visible between uterus (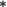) and ovary. (b) T2‐weighted axial magnetic resonance image, showing bilateral hematosalpinx (white arrows) and bilateral endometriomas (O). Adhesion between uterus (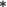), adnexa and rectum (R) is indicated by dashed circle. Rectal deep endometriosis is also visible (black arrow). (c) Intraoperative photograph depicting left ovarian endometrioma (O) and left hydrosalpinx (white arrow) lateral (Lat) to uterus (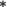). Med, medial.

The tubo‐ovarian condition (#Enzian compartment T) describes tubal patency and adhesions between ovaries, tubes and their surroundings. Ovarian immobility, a dynamic feature described in the IDEA approach as a ‘soft marker’, was assessed in all patients^14^. Severity was graded based on the extent of the adhesion as follows: Grade 0, no hydrosalpinx or adhesion; Grade 1, adhesions between the ovary and the pelvic sidewall with or without tubo‐ovarian adhesions; Grade 2, isolated adhesions between the adnexa and uterus, or Grade 1 plus adhesions to the uterus; and Grade 3, isolated adhesions between the adnexa and the uterosacral ligament (USL) and/or bowel, or Grade 2 plus adhesions to the USL and/or bowel. Tubal patency can be annotated with ‘+’ (patent) or ‘–’ (not patent). If hydrosalpinx was present, it was rated as non‐patent and classified as Grade 3 (Figure 1a).

DE lesions are typically hypoechogenic solid nodules. They can vary in size and have irregular or smooth contours. Pelvic DE occurs most commonly in the posterior compartment, as described in the IDEA consensus statement^3^. In the #Enzian classification, the posterior compartment is subdivided into three compartments: A, B and C. DE lesions were measured in three orthogonal planes. Based on the maximum diameter of the lesion in each compartment, a severity grade was assigned as follows: Grade 0, no lesion; Grade 1, < 1 cm; Grade 2, 1–3 cm; and Grade 3, > 3 cm.

Compartment A runs in the craniocaudal direction and assesses the posterior vaginal fornix, infiltration of the rectovaginal septum (RVS) and involvement of torus uterinus (the thickening at the site of insertion of the USL, behind the retrocervical area)^15^.

Compartment B runs in the mediolateral direction and assesses the USL, cardinal ligament, lateral parametrium and pelvic sidewall^16^, ^17^.

**Figure 2:**
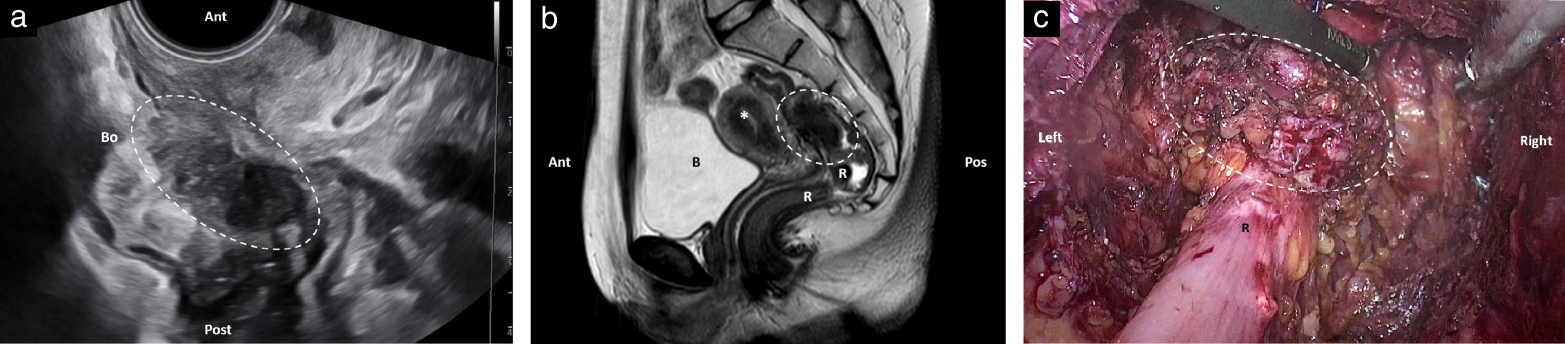
Endometriosis in #Enzian compartment C (rectosigmoid bowel) in patient undergoing surgery. (a) Transvaginal ultrasound image in left parasagittal plane, with probe in posterior vaginal fornix. Typical ‘moose antler’‐shaped hypoechogenic deep endometriotic (DE) nodule (dashed circle) infiltrates muscular layer and bulges into lumen of bowel (Bo). (b) T2‐weighted midsagittal magnetic resonance image of pelvis. Behind bladder (B) and uterus (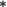), rectal DE (dashed circle) is seen with ‘mushroom cap’ appearance, with a shape similar to that on ultrasound in the anterior rectal wall. Rectum is indicated (R). (c) Intraoperative photograph showing rectum (R) and DE nodule (dashed circle) in anterior wall after complete dissection of pararectal spaces. Ant, anterior; Pos, posterior.

Compartment C includes the anterior wall of the rectum, whose course is marked by two major flexures in the anteroposterior axis. Characteristic signs for bowel endometriosis detailed in the IDEA consensus statement were used to identify rectal involvement^3^. DE lesions located up to the height of the uterine fundus (16 cm from the anal verge) were assigned to compartment C (Figure 2a). As per the IDEA approach, multifocal rectal DE lesions were classified according to the total length of the bowel segment involved, from caudal to cephalic aspect^18^, ^19^.

Further #Enzian localizations for DE lesions included: urinary bladder involvement (FB), ureteric involvement (FU), intestinal involvement (FI) and other extragenital locations (FO). There is no severity grading for lesions in these compartments according to the #Enzian classification.

#Enzian compartment FB included linear or spherical DE lesions infiltrating the muscularis layer or the submucosa in any part of the urinary bladder (trigonal zone, base, bladder dome, extra‐abdominal bladder) (Figure 3a). If only the serosa was affected, it was considered superficial disease. We also used the vesicouterine sliding sign for differential diagnosis and assessment of adhesions in this area^20^.

**Figure 3:**
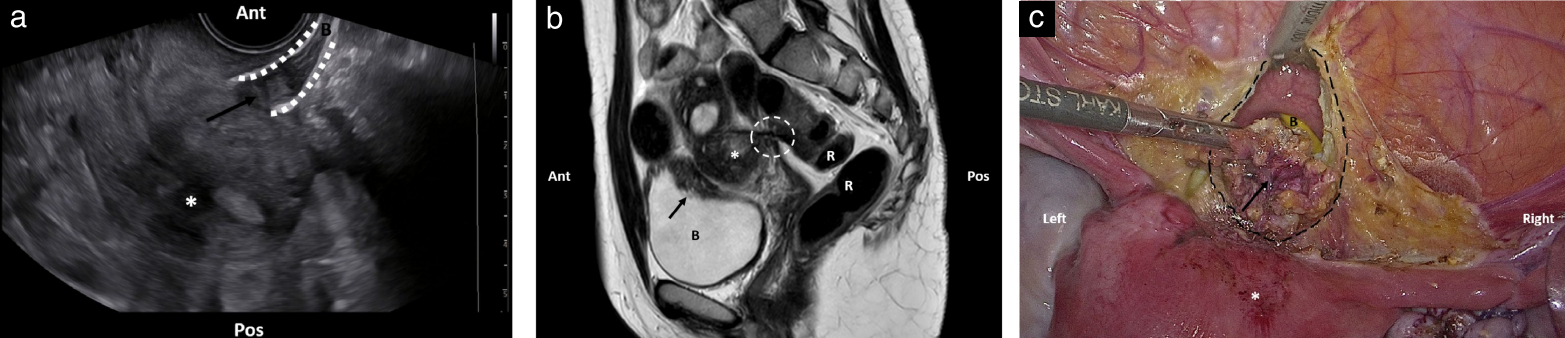
Endometriosis in #Enzian compartment FB (urinary bladder) in patient undergoing surgery. (a) Transvaginal ultrasound image in midsagittal plane, with transducer in anterior vaginal fornix. Anteflected adenomyotic uterus (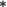) is adhered to almost empty bladder (B). Contour of hyperechogenic vesicovaginal septum and vesicouterine pouch (white dotted lines) is interrupted by hypoechogenic deep endometriotic (DE) nodule (arrow) infiltrating dome of bladder. (b) T2‐weighted parasagittal magnetic resonance image of pelvis. Bladder is moderately filled. Inhomogeneous nodular bladder‐wall thickening involving muscular layer at anterior dome is visible (arrow). Concomitant large DE nodule (dashed circle) occupies rectouterine space between uterus (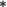) and rectum (R), with direct infiltration of rectal wall. (c) Intraoperative photograph showing opened urinary bladder (B). At height of bladder dome (dashed line), DE nodule (arrow) protrudes into cavity. Note adenomyotic uterus (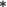) adhered to urinary bladder. Ant, anterior; Pos, posterior.

#Enzian compartment FU included both extrinsic ureteral DE lesions (i.e. lesions that compress the ureter from the outside) and intrinsic ureteral DE lesions (i.e. infiltration of the ureteral wall)^21^. Ureters were identified as hypoechogenic tubular structures, with a thick hyperechogenic wall, extending from the lateral aspect of the bladder base towards the pelvic sidewall.

#Enzian compartment FI included intestinal DE lesions above the rectosigmoid junction (above the level of the uterine fundus), as well as DE lesions in the sigmoid colon, appendix, other parts of the colon and small intestine. If there were multiple lesions in the same bowel segment, they were considered only for one #Enzian compartment (C or FI), while multicentric lesions affecting both the rectum and other bowel segments were considered for both C and FI compartments.

Lesions in the #Enzian compartment FO were classified according to their precise localization, e.g. F(sacral plexus), F(abdominal wall), F(diaphragm)^22^, ^23^, ^24^.

### MRI examination

MRI examinations were performed using a 1.5‐Tesla (T) machine (Philips Ingenia 1.5 T; Philips Healthcare, Best, The Netherlands) with a phased‐array pelvic coil. Bowel preparation with bisacodyl rectal suppositories and intravenous administration of an antiperistaltic agent (butylscopolamine) were performed in all cases. Patients were encouraged to empty their bladder approximately 1 h prior to the examination. No vaginal or rectal opacification was performed. Our acquisition protocol, including sequences and parameters, was based on ESUR guidelines^4^ and is detailed in Table S1. Gadolinium‐based contrast agent was administered routinely.

MRI data were reviewed using a Picture Archiving and Communication System workstation (Philips IntelliSpace Concerto; Philips Healthcare). Two radiologists (I.K., K.T.) with 25 and 5 years of experience (more than 5000 examinations), respectively, in female pelvic MRI interpreted the images using a structured reporting system^25^. Final interpretations were reached by consensus. Pelvic endometriosis was diagnosed according to imaging criteria described previously^5^, ^26^. The #Enzian score was routinely assigned and applied in clinical practice, whereas the deep pelvic endometriosis index and Endo‐Stage MRI scores were used solely for research purposes^27^.

T2‐weighted sequences without fat suppression served as the primary sequences for detecting pelvic DE. On T2‐weighted MRI, DE appears typically as irregular, linear, stellate or nodular areas of low signal intensity relative to muscle, reflecting fibrosis and stromal tissue. Cystic components may be variably present. Recurrent hemorrhage, inflammation and fibrosis may distort pelvic anatomy, leading to midline displacement of the uterus and abnormal ovarian positioning. Additional findings may include non‐visualization of the anterior or posterior cul‐de‐sac, loss of the typical low‐signal fibromuscular stroma of the cervix or vaginal wall and abrupt angulation of the bowel toward a focal pelvic lesion. Fat‐suppressed T1‐weighted sequences were used to detect hemorrhagic foci and peritoneal endometriosis^5^.

Adhesions around the ovaries and Fallopian tubes (#Enzian compartment T) were identified on MRI via direct visualization or secondary morphological signs (Figure 1b). Literature on the MRI appearance of pelvic adhesions is scarce; however, our approach is consistent with that taken by other authors^28^. Direct visualization of pelvic adhesions was defined as the presence of spiculated fibrous strands obscuring normal organ contours, demonstrating an isointense to mildly hypointense signal on T1‐weighted images and a low signal on T2‐weighted images. Secondary morphological signs included distortion of anatomical relationships and organ contours. An #Enzian severity score was assigned based on the location and extent of visible adhesions around the ovaries. For cases in which adhesions were not visualized directly, the severity score was estimated based on distortion of ovarian positioning relative to the pelvic sidewall, uterus, bowel and USL.

Ovarian endometrioma (#Enzian compartment O) was characterized on MRI as unilateral or bilateral, unilocular or multilocular cystic lesions exhibiting high signal intensity on fat‐suppressed T1‐weighted sequences, often accompanied by T2 shading and, occasionally, the ‘T2 dark spot’ sign.

Lesions in #Enzian compartment A typically appeared on MRI as T2‐hypointense, irregular, stellate or nodular lesions, with or without cystic components^26^.

Involvement of the USL (#Enzian compartment B) remains challenging to diagnose on MRI, as it often appears as non‐specific smooth or irregular thickening. A standardized definition proposed by the ENDOVALIRM group considers definitive USL involvement as abnormality visible in two imaging planes with the following appearance: spiculated (with retraction), nodular and/or irregular or smooth but with thickness > 5 mm, with or without hemorrhagic foci; or as any thickness in the presence of hemorrhagic implant^29^. This definition has been applied routinely in our clinical practice since its publication. Parametrial endometriosis is visualized typically as a T2‐hypointense area, sometimes with small T1‐hyperintense foci, in the paracervical or paravaginal region^30^.

Rectal and sigmoid involvement appears typically as crescent‐shaped, T2‐hypointense lesions of varying sizes. Lesions at the rectosigmoid junction often exhibit the characteristic ‘mushroom cap’ sign (Figure 2b), reflecting muscularis propria hypertrophy and adventitial retraction. The submucosal layer, appearing hyperintense on T2‐weighted imaging, may be thickened, representing non‐specific inflammation^31^. As per the #Enzian system, lesions located more than 16 cm from the anal verge are classified as compartment FI. However, the original publication does not specify which lesion component should exceed this level or how the measurement should be performed, limiting clinical applicability. In our study, lesions with any portion located less than 16 cm from the anal verge, measured along a curved axis, were assigned to #Enzian compartment C. Those extending above the anal verge were also classified as FI. While current consensus guidelines on endometriosis do not specify the measurement technique, they recommend assessment of the distance between the lesion and the anal verge^32^. The curved‐caliper technique has been described as providing accurate measurements and may represent a future standard for this assessment^31^.

Urinary bladder endometriosis (#Enzian compartment FB) appears typically on T2‐weighted MRI as a hypointense nodule, often associated with hyperintensity on fat‐saturated T1‐weighted sequences. These lesions are commonly situated at the level of the vesicouterine pouch, involving the bladder base or the bladder dome. They often form a broad‐based contact with the bladder wall and infiltrate the muscularis layer, demonstrated by disruption of the normal hypointense bladder‐wall signal on T2‐weighted imaging or protrusion into the bladder lumen in the case of mucosal involvement (Figure 3b). Ureteral endometriosis (#Enzian compartment FU) manifests typically as a T2‐hypointense nodule, frequently accompanied by retractile fibrotic adhesions involving surrounding adipose tissue^26^. Lesions were also reported in rare locations (e.g. sacral nerves, abdominal wall, appendix) (#Enzian compartment FO).

Although not analyzed in the current study, the presence of adenomyosis (#Enzian compartment FA) was reported in the MRI evaluation. The diagnosis was based on established direct and indirect imaging criteria^33^.

### Laparoscopy and surgical resection

Surgery was performed in a multidisciplinary set‐up, with the contribution of a surgeon (A.B.) experienced in minimally invasive gynecological surgery and the assistance of a colorectal surgeon, if needed. A nerve‐and‐vessel‐sparing technique was used during all procedures in order to decrease the prevalence of bladder and bowel functional sequelae^34^, ^35^. The surgical plan was tailored to the signs and symptoms of each patient. The primary objective during surgery was to achieve a visibly complete elimination of all endometriotic lesions, regardless of the technique used (Figures 1c, 2c and 3c). The location, size and extent of all pelvic DE lesions were classified intraoperatively using the #Enzian classification system and revised American Society for Reproductive Medicine (rASRM) score^7^, ^8^, ^9^.

### Statistical analysis

Continuous variables are expressed as mean ± SD and categorical variables are expressed as *n* (%). Sensitivity, specificity, positive (LR+) and negative (LR–) likelihood ratios, positive (PPV) and negative (NPV) predictive values and accuracy, with 95% CIs, for the detection of endometriotic lesions/adhesions by preoperative US and MRI were calculated using an online statistical calculator (https://www.medcalc.org/calc/diagnostic_test.php), with surgical evaluation considered as the reference standard. Sensitivity and specificity of US and MRI were compared using McNemar's test for paired proportions. *P* < 0.05 was considered statistically significant. The power of McNemar's test was analyzed using Statistica 14 software (Cloud Software Group Inc., Palo Alto, CA, USA).

## RESULTS

A total of 632 consecutive patients were primarily considered potentially eligible for inclusion (Figure S1). Of these, 144 opted for medical therapy or declined surgery, 20 underwent only one mode of imaging preoperatively (US or MRI) and another five who had opted for surgery did not give consent to participate in the study. The remaining 463 women who underwent laparoscopy for diagnosis and excisional treatment of endometriosis were enrolled in the study.

Demographic and clinical characteristics of the patients are displayed in Table 1. Comparison of sensitivity and specificity of US *vs* MRI for endometriotic lesions/adhesions in each #Enzian compartment, with 95% CIs, using McNemar's test, is presented in Table 2. The corresponding PPV, NPV, LR+, LR− and accuracy, with 95% CIs, for all #Enzian compartments are given in Table 3.

**Table 1:** Demographic and clinical characteristics of 463 women who underwent laparoscopy for clinical suspicion of endometriosis

Characteristic	Value
Age (years)	31 ± 5
Body mass index (kg/m^2^)	23 ± 6
Gravidity	
0	287 (62)
1	125 (27)
2	41 (9)
≥ 3	10 (2)
Parity	
0	342 (74)
1	74 (16)
2	39 (8)
≥ 3	8 (2)
Preoperative symptoms	
Dysmenorrhea	434 (94)
Dyschezia	117 (25)
Dysuria	75 (16)
Dyspareunia	235 (51)
Chronic pelvic pain	208 (45)
Infertility	214 (46)
Constipation	56 (12)
Diarrhea	51 (11)
Rectal bleeding	37 (8)
Hematuria	24 (5)
No endometriosis at laparoscopy	26 (6)
rASRM Stage	
1	29/437 (7)
2	65/437 (15)
3	198/437 (45)
4	145/437 (33)
History of pelvic surgery	
None	339 (73)
≥ 1 operation	124 (27)

Data are given as mean ± SD, *n* (%) or *n/N* (%). rASRM, revised American Society for Reproductive Medicine classification.

**Table 2:** Head‐to‐head comparison of sensitivity and specificity for detection of endometriotic lesions/adhesions in different #Enzian compartments by preoperative ultrasonography (US) *vs* magnetic resonance imaging (MRI)

	Sensitivity (%)				Specificity (%)			
#Enzian compartment	US	MRI	Difference	*P* *	US	MRI	Difference	*P* *
O_left_	97.52 (92.93–99.49)	95.04 (89.52–98.16)	2.48 (−2.36 to 7.32)	0.5078	96.77 (94.30–98.38)	95.89 (93.21–97.74)	0.88 (−1.99 to 3.75)	0.6900
O_right_	97.22 (92.10–99.42)	94.44 (88.30–97.93)	2.78 (−2.64 to 8.20)	0.5078	97.73 (95.58–99.02)	95.75 (93.09–97.6)	1.98 (0.55 to 4.52)	0.1892
T_left_	85.15 (76.69–91.44)	77.23 (67.82–84.98)	7.92 (−2.23 to 18.07)	0.1849	96.63 (94.19–98.25)	92.70 (89.48–95.17)	3.93 (0.56 to 7.30)	0.0336
T_right_	85.26 (76.51–91.70)	74.74 (64.78–83.09)	10.53 (0.22 to 20.83)	0.0755	94.79 (91.99–96.84)	89.86 (86.30–92.76)	4.93 (1.17 to 8.69)	0.0153
A	92.66 (88.35–95.75)	90.37 (85.65–93.94)	2.29 (−3.02 to 7.60)	0.4996	91.02 (86.72–94.29)	93.47 (89.61–96.22)	−2.45 (−7.24 to 2.34)	0.4050
B_left_	88.81 (82.21–93.60)	88.06 (81.33–93.02)	0.75 (−6.27 to 7.76)	> 0.9999	96.66 (94.10–98.32)	96.05 (93.34–97.88)	0.61 (−2.19 to 3.40)	0.8318
B_right_	86.79 (78.83–92.59)	90.57 (83.33 − 95.38)	−3.77 (−11.59 to 4.04)	0.4807	96.92 (94.55–98.45)	94.96 (92.15–96.98)	1.96 (−0.88 to 4.81)	0.2478
C	95.79 (91.87–98.16)	94.21 (89.88–97.07)	1.58 (−2.91 to 6.07)	0.6476	95.60 (92.45–97.71)	94.14 (90.66–96.61)	1.47 (−2.33 to 5.26)	0.5716
FI	75.00 (61.63–85.61)	83.93 (71.67–92.38)	−8.93 (−24.00 to 6.15)	0.3593	98.77 (97.16–99.60)	97.05 (94.91–98.47)	1.72 (−0.26 to 3.70)	0.1435
FU	76.19 (52.83–91.78)	71.43 (47.82–88.72)	4.76 (−16.01 to 25.53)	> 0.9999	97.96 (96.17–99.06)	99.10 (97.70–99.75)	−1.13 (−2.73 to 0.46)	0.2668
FB	83.72 (69.30–93.19)	74.42 (58.83–86.48)	9.30 (−3.29 to 21.89)	0.2891	99.05 (97.58–99.74)	98.57 (96.92–99.47)	0.48 (−1.00 to 1.95)	0.7539
FO	60.00 (26.24–87.84)	90.00 (55.50–99.75)	−30.00 (−69.69 to 9.69)	0.3750	99.56 (98.41–99.95)	98.90 (97.44–99.64)	0.66 (−0.48 to 1.81)	0.4531

Data in parentheses are 95% CI.

* McNemar's test. A, vagina, rectovaginal septum and torus uterinus; B, uterosacral ligament, cardinal ligament, parametrium and pelvic sidewall; C, rectosigmoid bowel; FB, urinary bladder; FI, other intestinal locations; FO, other extragenital locations; FU, ureters; O, ovary; T, tubo‐ovarian unit.

**Table 3:** Positive (PPV) and negative (NPV) predictive values, positive (LR+) and negative (LR−) likelihood ratios and accuracy for detection of endometriotic lesions/adhesions in different #Enzian compartments by preoperative ultrasonography (US) *vs* magnetic resonance imaging (MRI)

	PPV (%)		NPV (%)		LR+		LR−		Accuracy (%)	
#Enzian compartment	US	MRI	US	MRI	US	MRI	US	MRI	US	MRI
O_left_	91.48 (85.71–95.05)	89.15 (83.09–93.22)	99.10 (97.29–99.70)	98.20 (96.15–99.17)	30.23 (16.89–54.11)	23.15 (13.84–38.73)	0.03 (0.01–0.08)	0.05 (0.02–0.11)	96.97 (94.97–98.33)	95.67 (93.39–97.34)
O_right_	92.91 (86.84–96.30)	87.16 (80.50–91.78)	99.14 (97.42–99.72)	98.26 (96.28–99.19)	42.90 (21.61–85.17)	22.23 (13.52–36.55)	0.03 (0.01–0.09)	0.06 (0.03–0.13)	97.61 (95.70–98.80)	95.45 (93.12–97.16)
T_left_	87.75 (80.33–92.63)	75.00 (67.12–81.51)	95.82 (93.49–97.34)	93.48 (90.92–95.36)	25.26 (14.40–44.32)	10.57 (7.20–15.54)	0.15 (0.10–0.25)	0.25 (0.17–0.35)	94.09 (91.52–96.07)	89.28 (86.07–91.96)
T_right_	81.04 (73.25–86.97)	65.81 (58.12–72.75)	96.10 (93.82–97.56)	93.16 (90.59–95.07)	16.38 (10.49–25.58)	7.37 (5.32–10.23)	0.16 (0.10–0.25)	0.28 (0.20–0.40)	92.82 (90.07–95.01)	86.73 (83.29–89.70)
A	90.18 (86.03–93.20)	92.49 (88.45–95.2)	93.30 (89.67–95.72)	91.60 (87.87–94.25)	10.32 (6.91–15.40)	13.84 (8.60–22.27)	0.08 (0.05–0.13)	0.10 (0.07–0.15)	91.79 (88.91–94.13)	92.01 (89.15–94.31)
B_left_	91.52 (85.76–95.09)	90.06 (84.12–93.94)	95.50 (92.95–97.16)	95.19 (92.59–96.91)	26.56 (14.81–47.63)	22.29 (13.03–38.11)	0.12 (0.07–0.19)	0.12 (0.08–0.20)	94.39 (91.88–96.30)	93.74 (91.13–95.77)
B_right_	89.32 (82.31–93.77)	84.21 (77.21–89.37)	96.11 (93.81–97.58)	97.13 (94.94–98.39)	28.17 (15.67–50.64)	17.96 (11.40–28.29)	0.14 (0.08–0.22)	0.10 (0.06–0.18)	94.60 (92.13–96.48)	93.95 (91.38–95.94)
C	93.81 (89.69–96.34)	91.78 (87.40–94.74)	97.03 (94.31–98.47)	95.90 (92.95–97.65)	21.79 (12.52–37.92)	16.07 (9.98–25.89)	0.04 (0.02–0.09)	0.06 (0.03–0.11)	95.68 (93.41–97.34)	94.17 (91.63–96.12)
FI	89.37 (77.64–95.32)	79.67 (68.93–87.38)	96.63 (94.80–97.83)	97.77 (96.02–98.76)	61.05 (25.22–147.80)	28.47 (16.11–50.29)	0.25 (0.16–0.40)	0.17 (0.09–0.30)	95.90 (93.66–97.51)	95.46 (93.15–97.17)
FU	63.81 (46.95–77.84)	78.81 (57.47–91.10)	98.87 (97.60–99.47)	98.66 (97.40–99.31)	37.42 (18.78–74.56)	78.93 (28.68–217.22)	0.24 (0.11–0.52)	0.29 (0.15–0.57)	96.98 (94.99–98.34)	97.85 (96.08–98.97)
FB	90.01 (77.11–96.02)	84.23 (70.31–92.34)	98.34 (96.79–99.15)	97.41 (95.76–98.43)	87.91 (32.85–235.21)	52.09 (23.09–117.51)	0.16 (0.08–0.32)	0.26 (0.16–0.43)	97.62 (95.79–98.81)	96.33 (94.18–97.84)
FO	75.35 (41.21–93.02)	64.72 (42.82–81.79)	99.10 (98.11–99.58)	99.77 (98.56–99.96)	135.90 (31.17–592.57)	81.54 (33.29–199.72)	0.40 (0.19–0.86)	0.10 (0.02–0.65)	98.69 (97.18–99.51)	98.70 (97.20–99.52)

Values in parentheses are 95% CI. A, vagina, rectovaginal septum and torus uterinus; B, uterosacral ligament, cardinal ligament, parametrium and pelvic sidewall; C, rectosigmoid bowel; FB, urinary bladder; FI, other intestinal locations; FO, other extragenital locations; FU, ureters; O, ovary; T, tubo‐ovarian unit.

Ovarian endometriosis (#Enzian compartment O) was diagnosed during laparoscopy in 229 patients: 121/462 (26.2%) on the left side (the left ovary had been removed previously in one patient) and 108/461 (23.4%) on the right side (the right ovary had been removed previously in two patients). All existing ovaries were visualized successfully. On the left side, endometrioma was identified correctly in 118/121 (97.5%) patients by US and 115/121 (95.0%) patients by MRI. On the right side, endometrioma was identified correctly in 105/108 (97.2%) patients by US and 102/108 (94.4%) patients by MRI. On both sides, both US and MRI had high sensitivity (left side, 97.52% *vs* 95.04%, *P* = 0.5078; right side, 97.22% *vs* 94.44%, *P* = 0.5078) and high specificity (left side, 96.77% *vs* 95.89%, *P* = 0.6900; right side, 97.73% *vs* 95.75%, *P* = 0.1892). The false‐positive cases (11 on the left side and eight on the right side with US; 14 on the left side and 15 on the right side with MRI) were hemorrhagic functional cysts or corpus luteum cysts.

Endometriosis in the tubo‐ovarian unit (#Enzian compartment T) was diagnosed during laparoscopy in 196 patients: 101/457 (22.1%) on the left side (the left tube had been removed previously in six patients) and 95/460 (20.7%) on the right side (the right tube had been removed previously in three patients). All existing tubes were assessed successfully. On the left side, endometriosis was identified correctly in 86/101 (85.1%) patients by US and 78/101 (77.2%) patients by MRI. On the right side, endometriosis was identified correctly in 81/95 (85.3%) patients by US and 71/95 (74.7%) patients by MRI. On both sides, US showed significantly higher specificity compared with MRI (left side, 96.63% *vs* 92.70%, *P* = 0.0336; right side, 94.79% *vs* 89.86%, *P* = 0.0153), as well as non‐significantly higher sensitivity (left side, 85.15% *vs* 77.23%, *P* = 0.1849; right side, 85.26% *vs* 74.74%, *P* = 0.0755).

DE in #Enzian compartment A was diagnosed during laparoscopy in 218/463 (47.1%) patients. DE was identified correctly in 202/218 (92.7%) patients by US and 197/218 (90.4%) patients by MRI. Both US and MRI showed high sensitivity (92.66% *vs* 90.37%; *P* = 0.4996) and high specificity (91.02% *vs* 93.47%; *P* = 0.4050).

DE in #Enzian compartment B was diagnosed in 240 patients: 134/463 (28.9%) on the left side and 106/463 (22.9%) on the right side. On the left side, DE was identified correctly in 119/134 (88.8%) patients by US and 118/134 (88.1%) patients by MRI. On the right side, DE was identified correctly in 92/106 (86.8%) patients by US and 96/106 (90.6%) patients by MRI. On both sides, US and MRI had similar sensitivity (left side, 88.81% *vs* 88.06%, *P* > 0.9999; right side, 86.79% *vs* 90.57%, *P* = 0.4807) and similar specificity (left side, 96.66% *vs* 96.05%, *P* = 0.8318; right side, 96.92% *vs* 94.96%, *P* = 0.2478).

DE in #Enzian compartment C was diagnosed during laparoscopy in 190/463 (41.0%) patients. DE was identified correctly in 182/190 (95.8%) patients by US and 179/190 (94.2%) patients by MRI. Both US and MRI showed similarly high sensitivity (95.79% *vs* 94.21%; *P* = 0.6476) and high specificity (95.60% *vs* 94.14%; *P* = 0.5716).

**Figure 4:**
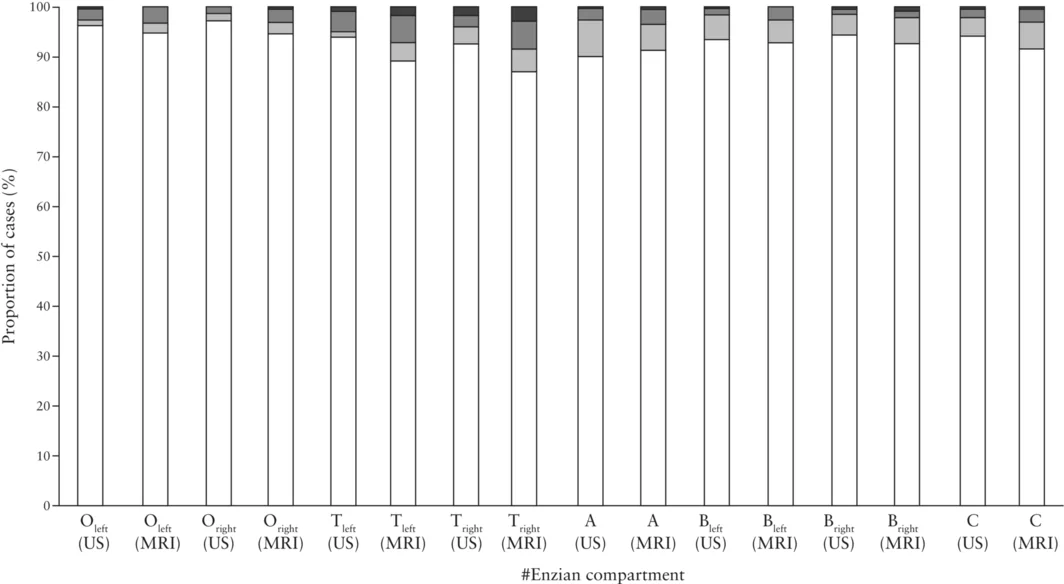
Concordance between preoperative imaging and surgical assessment in #Enzian severity grade of endometriotic lesions/adhesions in #Enzian compartments O, T, A, B and C. 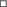, no difference in severity grade; 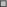, difference of one severity grade; 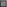, difference of two severity grades; 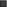, difference of three severity grades. It should be noted that, for example, a difference of two severity grades in this representation may be due to a Grade‐2 lesion/adhesion being missed on preoperative imaging or a Grade‐2 lesion/adhesion being seen on imaging that could not be detected at surgery, as well as due to classification of the severity of a lesion/adhesion as Grade 1 on imaging and Grade 3 at surgery or *vice versa*. A, vagina, rectovaginal septum and torus uterinus; B, uterosacral ligament, cardinal ligament, parametrium and pelvic sidewall; C, rectosigmoid bowel; MRI, magnetic resonance imaging; O, ovary; T, tubo‐ovarian unit; US, ultrasonography.

The concordance between preoperative imaging and surgical assessment in the grading of severity for endometriotic lesions/adhesions in #Enzian compartments O, T, A, B and C are shown in Tables 4 and S2 and Figures 4 and S2. Regarding assessment of the adnexa (#Enzian compartments O and T), US showed higher exact concordance on both sides in both compartments compared with MRI: O_left_, 96.3% *vs* 94.8%; O_right_, 97.2% *vs* 94.6%; T_left_, 93.9% *vs* 89.0%; T_right_, 92.6% *vs* 86.7%. When examining DE nodules, US showed 3.1 percentage points higher exact concordance compared with MRI in #Enzian compartment C (94.0% *vs* 90.9%). In #Enzian compartments A and B, the rates of concordance were also above 90.0%, and the differences between imaging modalities were less than 2 percentage points.

**Table 4:** Concordance between preoperative imaging and surgical assessment of #Enzian severity grade for endometriotic lesions/adhesions in #Enzian compartments O, T, A, B and C

	Severity on preoperative US				Severity on preoperative MRI			
#Enzian compartment/ severity at surgical assessment	Grade 0	Grade 1	Grade 2	Grade 3	Grade 0	Grade 1	Grade 2	Grade 3
O_left_ (*n* = 462)								
Grade 0	330 (71.4)	2 (0.4)	8 (1.7)	1 (0.2)	327 (70.8)	4 (0.9)	10 (2.2)	0 (0)
Grade 1	0 (0)	24 (5.2)	0 (0)	0 (0)	1 (0.2)	23 (5.0)	0 (0)	0 (0)
Grade 2	3 (0.6)	0 (0)	77 (16.7)	1 (0.2)	5 (1.1)	0 (0)	73 (15.8)	3 (0.6)
Grade 3	0 (0)	0 (0)	2 (0.4)	14 (3.0)	0 (0)	0 (0)	1 (0.2)	15 (3.2)
O_right_ (*n* = 461)								
Grade 0	345 (74.8)	3 (0.7)	5 (1.1)	0 (0)	338 (73.3)	6 (1.3)	8 (1.7)	1 (0.2)
Grade 1	2 (0.4)	13 (2.8)	0 (0)	0 (0)	1 (0.2)	12 (2.6)	2 (0.4)	0 (0)
Grade 2	1 (0.2)	1 (0.2)	68 (14.8)	0 (0)	4 (0.9)	1 (0.2)	64 (13.9)	1 (0.2)
Grade 3	0 (0)	0 (0)	1 (0.2)	22 (4.8)	1 (0.2)	0 (0)	0 (0)	22 (4.8)
T_left_ (*n* = 457)								
Grade 0	344 (75.3)	3 (0.7)	8 (1.8)	1 (0.2)	330 (72.2)	7 (1.5)	16 (3.5)	3 (0.7)
Grade 1	1 (0.2)	25 (5.5)	1 (0.2)	0 (0)	9 (2.0)	18 (3.9)	0 (0)	0 (0)
Grade 2	11 (2.4)	0 (0)	37 (8.1)	0 (0)	9 (2.0)	0 (0)	39 (8.5)	0 (0)
Grade 3	3 (0.7)	0 (0)	0 (0)	23 (5.0)	5 (1.1)	0 (0)	1 (0.2)	20 (4.4)
T_right_ (*n* = 460)								
Grade 0	346 (75.2)	11 (2.4)	6 (1.3)	2 (0.4)	328 (71.3)	16 (3.5)	15 (3.3)	6 (1.3)
Grade 1	4 (0.9)	21 (4.6)	0 (0)	0 (0)	6 (1.3)	19 (4.1)	0 (0)	0 (0)
Grade 2	4 (0.9)	0 (0)	39 (8.5)	1 (0.2)	11 (2.4)	0 (0)	33 (7.2)	0 (0)
Grade 3	6 (1.3)	0 (0)	0 (0)	20 (4.3)	7 (1.5)	0 (0)	0 (0)	19 (4.1)
A (*n* = 463)								
Grade 0	223 (48.2)	13 (2.8)	9 (1.9)	0 (0)	229 (49.5)	8 (1.7)	8 (1.7)	0 (0)
Grade 1	13 (2.8)	127 (27.4)	6 (1.3)	0 (0)	13 (2.8)	130 (28.1)	3 (0.6)	0 (0)
Grade 2	2 (0.4)	0 (0)	48 (10.4)	0 (0)	6 (1.3)	0 (0)	43 (9.3)	1 (0.2)
Grade 3	1 (0.2)	0 (0)	2 (0.4)	19 (4.1)	2 (0.4)	0 (0)	2 (0.4)	18 (3.9)
B_left_ (*n* = 463)								
Grade 0	318 (68.7)	8 (1.7)	3 (0.6)	0 (0)	316 (68.3)	7 (1.5)	6 (1.3)	0 (0)
Grade 1	11 (2.4)	35 (7.6)	1 (0.2)	0 (0)	10 (2.2)	34 (7.3)	3 (0.6)	0 (0)
Grade 2	3 (0.6)	0 (0)	68 (14.7)	3 (0.6)	6 (1.3)	1 (0.2)	67 (14.5)	0 (0)
Grade 3	1 (0.2)	0 (0)	0 (0)	12 (2.6)	0 (0)	0 (0)	0 (0)	13 (2.8)
B_right_ (*n* = 463)								
Grade 0	346 (74.7)	7 (1.5)	3 (0.6)	1 (0.2)	339 (73.2)	13 (2.8)	4 (0.9)	1 (0.2)
Grade 1	11 (2.4)	39 (8.4)	0 (0)	0 (0)	5 (1.1)	43 (9.3)	2 (0.4)	0 (0)
Grade 2	2 (0.4)	0 (0)	42 (9.1)	1 (0.2)	2 (0.4)	2 (0.4)	39 (8.4)	2 (0.4)
Grade 3	1 (0.2)	0 (0)	0 (0)	10 (2.2)	3 (0.6)	0 (0)	0 (0)	8 (1.7)
C (*n* = 463)								
Grade 0	261 (56.4)	7 (1.5)	5 (1.1)	0 (0)	257 (55.5)	8 (1.7)	8 (1.7)	0 (0)
Grade 1	3 (0.6)	55 (11.9)	6 (1.3)	0 (0)	4 (0.9)	54 (11.7)	5 (1.1)	1 (0.2)
Grade 2	3 (0.6)	0 (0)	83 (17.9)	1 (0.2)	5 (1.1)	1 (0.2)	76 (16.4)	5 (1.1)
Grade 3	2 (0.4)	0 (0)	1 (0.2)	36 (7.8)	2 (0.4)	0 (0)	3 (0.6)	34 (7.3)

Data are given as *n* (%). Severity grading for ovarian (O) endometriosis: Grade 0, no lesion; Grade 1, sum of maximum diameters of lesions < 3 cm; Grade 2, sum of maximum diameters of lesions 3–7 cm; Grade 3, sum of maximum diameters of lesions > 7 cm. Severity grading for tubo‐ovarian condition (T): Grade 0, no hydrosalpinx or adhesion; Grade 1, adhesions between ovary and pelvic sidewall with or without tubo‐ovarian adhesions; Grade 2, isolated adhesions between adnexa and uterus, or Grade 1 plus adhesions to uterus; Grade 3, hydrosalpinx or isolated adhesions between adnexa and uterosacral ligament (USL) and/or bowel, or Grade 2 plus adhesions to USL and/or bowel. Severity grading for deep endometriosis in posterior compartment: Grade 0, no lesion; Grade 1, maximum diameter of lesion < 1 cm; Grade 2, maximum diameter of lesion 1–3 cm; Grade 3, maximum diameter of lesion > 3 cm. A, vagina, rectovaginal septum and torus uterinus; B, uterosacral ligament, cardinal ligament, parametrium and pelvic sidewall; C, rectosigmoid bowel; MRI, magnetic resonance imaging; US, ultrasonography.

For #Enzian compartments FI, FB, FU and FO, for which there is no distinction in severity grade according to the #Enzian classification, the concordance between preoperative imaging and surgical assessment in the detection of endometriotic lesions is shown in Table 5. DE in the cranially located bowel segments (#Enzian compartment FI) was diagnosed during laparoscopy in 56/463 (12.1%) patients. DE was identified correctly in 42/56 (75.0%) patients by US and 47/56 (83.9%) patients by MRI. There was a trend towards higher sensitivity for MRI compared with US (83.93% *vs* 75.00%), however the difference between modalities was not statistically significant for sensitivity (*P* = 0.3593) or specificity (*P* = 0.1435). Among the 56 patients with DE in #Enzian compartment FI, 48 had DE in the sigmoid colon (40/48 diagnosed by US and 43/48 diagnosed by MRI), five had DE in the appendix (1/5 diagnosed by US and 2/5 diagnosed by MRI), two had DE in the cecum (0/2 diagnosed by US and 2/2 diagnosed by MRI) and one had DE in the ileum (1/1 diagnosed by US and 0/1 diagnosed by MRI).

**Table 5:** Concordance between preoperative imaging and surgical assessment in detection of endometriotic lesions #Enzian compartments FB, FU, FI and FO (*n* = 463)

	Preoperative US		Preoperative MRI	
#Enzian compartment/ surgical assessment	Lesion	No lesion	Lesion	No lesion
FB				
Lesion	36 (7.8)	7 (1.5)	32 (6.9)	11 (2.4)
No lesion	4 (0.9)	416 (89.8)	6 (1.3)	414 (89.4)
FU				
Lesion	16 (3.5)	5 (1.1)	15 (3.2)	6 (1.3)
No lesion	9 (1.9)	433 (93.5)	4 (0.9)	438 (94.6)
FI				
Lesion	42 (9.1)	14 (3.0)	47 (10.2)	9 (1.9)
No lesion	5 (1.1)	402 (86.8)	12 (2.6)	395 (85.3)
FO				
Lesion	6 (1.3)	4 (0.9)	9 (1.9)	1 (0.2)
No lesion	2 (0.4)	451 (97.4)	5 (1.1)	448 (96.8)

Data are given as *n* (%). FB, urinary bladder; FI, other intestinal locations; FO, other extragenital locations; FU, ureters; MRI, magnetic resonance imaging; US, ultrasonography.

Among the remaining #Enzian compartments, bladder endometriosis (#Enzian compartment FB) was the most common, with a prevalence of 9.3% (43/463). DE was identified correctly in 36/43 (83.7%) patients by US and 32/43 (74.4%) patients by MRI. The sensitivity of US and MRI for bladder DE (83.72% *vs* 74.42%; *P* = 0.2891) was lower in this location compared with that in the rectum. However, both modalities showed high specificity for bladder DE (99.05% *vs* 98.57%; *P* = 0.7539).

Ureteral DE (#Enzian compartment FU) was found during laparoscopy in 21/463 (4.5%) patients. Ureteral lesions were left‐sided in 13/21 (61.9%) patients and right‐sided in 8/21 (38.1%) patients. Of the 21 cases, 15 (71.4%) had intrinsic‐type ureteral DE lesions on histological assessment. Ureteral DE was identified correctly in 16/21 (76.2%) patients by US and 15/21 (71.4%) patients by MRI. Both US and MRI showed lower sensitivity for ureteral DE (76.19% *vs* 71.43%; *P* > 0.9999) compared with bladder DE, but specificities were similarly high (97.96% *vs* 99.10%; *P* = 0.2668).

DE in any other location (#Enzian compartment FO) was found during laparoscopy in 10/463 (2.2%) patients, including three cases in the abdominal wall, four cases in the sacral plexus and three cases in the diaphragm. DE in these locations was identified correctly in 6/10 (60.0%) patients by US and 9/10 (90.0%) patients by MRI. US missed all three diaphragmatic cases and one case of abdominal‐wall DE; MRI could not identify one case of sacral plexus DE.

## DISCUSSION

Timely confirmation of the presence of endometriosis and accurately mapping its extent offer numerous advantages. A correct preoperative diagnosis not only avoids an unexpected intraoperative scenario, but also helps to provide patients with accurate information and organize an appropriate surgical team. Endometriosis can occur in many forms and locations. Determining which diagnostic method is most effective in each anatomical compartment has been a challenging clinical question in recent years. To the best of our knowledge, our prospective, head‐to‐head comparative diagnostic accuracy study is the first to compare and analyze in detail the diagnostic efficacy of two different imaging modalities (US and MRI), according to localization and severity of endometriotic lesion/adhesion, with use of the #Enzian classification system.

An important finding of this study is that US has significantly higher specificity compared with MRI for the assessment of pelvic adhesions in #Enzian compartment T. Severe adhesions of the adnexa lateral to the pelvic sidewall, inferomedial to the parametrium or medial to the uterus or rectum, leading to complete obliteration of the pouch of Douglas, fundamentally determine a patient's symptoms and the extent of possible surgery. The dynamic nature of TVS examination may explain its superior specificity. Soft markers described in the IDEA protocol, such as direct feedback from the patient during the examination about site‐specific tenderness and assessment of ovarian immobility, are advantageous for the assessment of pelvic adhesions^3^, ^14^.

Both US and MRI offer high accuracy for diagnosing endometrioma. However, there is a paucity of high‐quality studies comparing these two imaging modalities. In the meta‐analysis of Kanti *et al*.^36^, MRI had higher specificity compared with TVS, but only two of the 14 included studies performed a direct comparison between modalities (57 endometriomas in 114 patients). In the present study of 463 patients (229 endometriomas), US had specificity and sensitivity ≥ 96.8% on both sides, whereas MRI had sensitivity and specificity ≥ 94.4% on both sides in #Enzian compartment O.

Studies conducted prior to the publication of the IDEA consensus statement reported ambiguous findings, but suggested that MRI could be more effective compared with US in the diagnosis of DE in certain pelvic locations^37^, ^38^. However, our results broadly support those reported in the pilot study of Indrielle‐Kelly *et al*.^39^, which showed that US examination using the IDEA approach has similarly high sensitivity and specificity in all pelvic compartments as MRI.

In their 2018 meta‐analysis, Guerriero *et al*.^12^ reported that the diagnostic performance of TVS is similar to that of MRI for detecting DE involving the USL and RVS. In contrast, Gerges *et al*.^40^ reported in 2021 that MRI outperformed TVS for the preoperative diagnosis of USL and vaginal DE in terms of sensitivity, although the specificity of both modalities was excellent. In a recent head‐to‐head comparative meta‐analysis focused on RVS involvement, Lou *et al*.^41^ found that MRI exhibited higher sensitivity and comparable specificity to TVS, although the authors cautioned that the limited number of articles may affect the robustness of these results. Our data support the conclusion of Ros *et al*.^42^ that TVS can be used as the first‐line diagnostic tool in #Enzian compartments A and B. In their study, US had a sensitivity of 87.0% for DE involving the posterior vaginal fornix and 83.9% for DE involving the torus uterinus, with respective specificities of 94.6% and 89.4%. Correspondingly, we report a sensitivity of 92.66% and a specificity of 91.02% for the detection by US of DE in #Enzian compartment A. Ros *et al*.^42^ found that the sensitivity and specificity of US for DE involving the USL were 86.1% and 74.2%, respectively, on the left side and 91.4% and 75.8%, respectively, on the right side. In the present study, the sensitivity and specificity of US for the detection of DE in #Enzian compartment B were 88.81% and 96.66%, respectively, on the left side and 86.79% and 96.92%, respectively, on the right side.

Both MRI and US are used for the assessment of rectosigmoid DE in #Enzian compartment C^19^, ^43^. In a 2018 study of 317 patients, Alborzi *et al*.^44^ found that TVS and transrectal US have adequate diagnostic accuracy for rectosigmoid DE, comparable with that of MRI. Guerriero *et al*.^11^ reported a statistically significant difference between two‐dimensional US and MRI on receiver‐operating‐characteristics‐curve analysis for the identification of intestinal DE, although differences in sensitivity and specificity were not significant on McNemar's test. In 2019, Moura *et al*.^45^ found that both MRI and TVS exhibit 90% sensitivity and 96% specificity in the diagnosis of rectosigmoid DE. In the present study population, sensitivity and specificity were both around 96% for US and 94% for MRI for DE in #Enzian compartment C. In this location, exact concordance in severity grade between preoperative imaging and surgical assessment was approximately 3 percentage points higher for US compared with MRI.

This study included 43 patients who underwent surgery for bladder endometriosis. Sensitivity was low compared with that for posterior pelvic compartment localizations for both imaging modalities (US, 83.72%; MRI, 74.42%), although specificity was above 98% for both. Gerges *et al*.^46^ reported similar results for US in their meta‐analysis examining the optimal diagnosis of bladder endometriosis; however, insufficient data were available for the evaluation of MRI.

MRI still appears to be more effective in assessing extrapelvic endometriotic lesions (e.g. diaphragm, cecum) compared with US, but, owing to their rare occurrence, no statistically significant difference in sensitivity or specificity was detected between imaging modalities in #Enzian compartment FO. Future studies should compare the effectiveness of US and MRI in the diagnosis of endometriosis in individual extrapelvic locations.

Strengths of our study include the large number of participants, the use of a consistent classification system by radiologists, sonographers and surgeons, and the fact that all imaging experts were blinded to preoperative clinical findings and each other's results. However, despite the large study population, the comparison of imaging modalities was underpowered for non‐inferiority claims in low‐prevalence locations, such as the ureter (#Enzian compartment FU) and extrapelvic sites (#Enzian compartment FO). Another limitation is that our study was conducted in a tertiary referral center for endometriosis, so our findings may not be generalizable. However, such a comparative study can only be performed at a center that employs qualified and experienced specialists in both imaging modalities^47^. Moreover, surgeons were not blinded to the US and MRI results. This lack of blinding may have led to more targeted surgical exploration in cases with positive preoperative imaging findings, even though the objective in every operation was to obtain a complete overview of pelvic anatomy and to remove all endometriotic nodules. This could have led to overestimation of the sensitivity of both imaging modalities. However, the indication for surgery was based on clinical symptoms prior to imaging examinations, and surgeons could use these results to provide appropriate care.

In conclusion, our study provides robust evidence that both US and MRI are equally effective in diagnosing DE. However, US demonstrated superior specificity compared with MRI for assessing pelvic adhesions in #Enzian compartment T.

## Supporting information


**Table S1** Parameters for magnetic resonance imaging sequences.

**Table S2** Exact concordance between preoperative imaging and surgical assessment of #Enzian severity grade for endometriotic lesions/adhesions in #Enzian compartments O, T, A, B and C.

**Figure S1** STARD flowchart summarizing inclusion of patients in study.

Green, no difference in severity grade; yellow, difference of ± 1 severity grade; red, difference of ± 2 severity grades; purple, difference of ± 3 severity grades. It should be noted that, for example, a difference of two severity grades in this representation may be due to a Grade‐2 lesion/adhesion being missed on preoperative imaging or a Grade‐2 lesion/adhesion being seen on imaging that could not be detected at surgery, as well as due to classification of a lesion/adhesion as Grade 1 on imaging and Grade 3 at surgery, or *vice versa*. A, vagina, rectovaginal septum and torus uterinus; B, uterosacral ligament, cardinal ligament, parametrium and pelvic sidewall; C, rectosigmoid bowel; MRI, magnetic resonance imaging; O, ovary; T, tubo‐ovarian unit; US, ultrasonography.

## Data Availability

The data that support the findings of this study are available on request from the corresponding author. The data are not publicly available due to privacy or ethical restrictions.
