# Supplementary material for: Head‐to‐head prospective comparison of diagnostic accuracy of preoperative ultrasonography and MRI in patients undergoing surgery for deep endometriosis using #Enzian classification
Source: Ultrasound Obstet Gynecol. 2026 Aug 6;68(3):413–25. doi: 10.1002/uog.70298 (PMC13532160; doi:10.1002/uog.70298)
Supplement: Supplementary file 1 [file UOG-68-413-s001.docx]

**Table S1** Parameters for magnetic resonance imaging sequences.

|  | **Axial T2W TSE*** | **Sagittal T2W TSE** | **Coronal T2W TSE** | **Axial T2W SPAIR** | **Axial T1W TSE** | **Axial T1 SPIR** | **Axial DWI**** | **Axial T1 SPIR + C** | **Sagittal T1 SPIR + C** |
| --- | --- | --- | --- | --- | --- | --- | --- | --- | --- |
| TE^ǂ^ (ms) | 100 | 100 | 90 | 80 | 10 | 10 | shortest | 10 | 10 |
| TR^†^ (ms) | 1000 | 3000-6000 | 3000-5000 | 3000-5000 | 400-600 | 400-600 | shortest | 400-700 | 400-700 |
| Flip angle(°) | 90 | 90 | 90 | 90 | 90 | 90 | 90 | 90 | 90 |
| Matrix (mm) | 280x214 | 264x201 | 280x244 | 268x268 | 240x290 | 240x290 | 124x90 | 240x285 | 252x215 |
| FOV^#^ AP (mm) | 345 | 250 | 145 | 240 | 240 | 240 | 266 | 240 | 250 |
| FOV RL (mm) | 390 | 250 | 300 | 328 | 332 | 333 | 375 | 333 | 145 |
| FOV FH (mm) | 193 | 145 | 250 | 191 | 191 | 191 | 191 | 191 | 250 |
| NSA | 1 | 2 | 2 | 1 | 1 | 1 | 3 | 1 | 1 |
| Bandwith (Hz) | 633.2 | 173.4 | 168.1 | 193.1 | 223.5 | 235.7 | 44.2 | 223.5 | 223.4 |
| Voxel size (mm) | 1 | 1 | 1 | 1 | 1 | 1 | 3 | 1 | 1 |
| Slice thickness (mm) | 4 | 4 | 4 | 4 | 4 | 4 | 4 | 4 | 4 |
| Acquisition time (min) | 0:45 | 2:50 | 1:40 | 1:30 | 1:30 | 2:30 | 2:00 | 1:40 | 3:20 |

NSAx, number of signal averages; FOV^#^: Field of view; TE^ǂ^: time of echo; TR^†^: time of repetition; DWI: Diffusion-weighted imaging;

SPAIR: Spectral Adiabatic Inversion Recovery; SPIR(+C): Spectral Presaturation with Inversion Recovery (with a contrast agent); TSE: Turbo Spin Echo;

* From renal hila to pubic symphysis.

** b values: 0, 400, 800.
