# Supplementary material for: Head‐to‐head prospective comparison of diagnostic accuracy of preoperative ultrasonography and MRI in patients undergoing surgery for deep endometriosis using #Enzian classification
Source: Ultrasound Obstet Gynecol. 2026 Aug 6;68(3):413–25. doi: 10.1002/uog.70298 (PMC13532160; doi:10.1002/uog.70298)
Supplement: Supplementary file 2 [file UOG-68-413-s003.docx]

**Table S2** Exact concordance between preoperative imaging and surgical assessment of #Enzian severity grade for endometriotic lesions/adhesions in #Enzian compartments O, T, A, B and C

| **#Enzian compartment** | **Preoperative US** | **Preoperative MRI** |
| --- | --- | --- |
| O_left_ | 96.3 | 94.8 |
| O_right_ | 97.2 | 94.6 |
| T_left_ | 93.9 | 89.0 |
| T_right_ | 92.6 | 86.7 |
| A | 90.1 | 90.8 |
| B_left_ | 93.6 | 92.9 |
| B_right_ | 94.4 | 92.6 |
| C | 94.0 | 90.9 |

Data are given as %. MRI, magnetic resonance imaging; US, ultrasonography.
